# Banhasasim-Tang Ameliorates Spatial Memory by Suppressing Oxidative Stress through Regulation of ERK/p38 Signaling in Hippocampus of Mice

**DOI:** 10.1155/2021/6970578

**Published:** 2021-12-02

**Authors:** Malk Eun Pak, You-Chang Oh, Yeo Jin Park, Jae Kwang Kim, Min-Gyeong Shin, Jong Hyuk Yoon, Younghoon Go

**Affiliations:** ^1^Korean Medicine- (KM-) Application Center, Korea Institute of Oriental Medicine, Daegu 41062, Republic of Korea; ^2^Korean Convergence Medicine, University of Science and Technology, Daejeon 34054, Republic of Korea; ^3^Neurodegenerative Diseases Research Group, Korean Brain Research Institute, Daegu 41062, Republic of Korea

## Abstract

Since ancient times, Banhasasim-tang (BHS) has been used to treat functional dyspepsia in East Asia. Here, we aimed to determine the protective action of BHS on hippocampal neurons against oxidative stress. We investigated the functional effect of BHS on a scopolamine-induced mouse model, and molecular analysis was performed in glutamate-induced HT22 cells. We observed that BHS administration ameliorated memory dysfunction in scopolamine-treated mice. BHS administration also increased neuronal survival and acetylcholine activity and phosphorylation of extracellular signal-regulated kinase (ERK) and cAMP response element-binding protein (CREB) in the hippocampus of mice. In hippocampal cells, BHS treatment rescued glutamate-induced cytotoxicity, apoptosis, and oxidative stress. We observed an increase of HO-1 and a decrease of Nrf2 protein expression in glutamate-induced oxidative stress; however, the expression level of these proteins was significantly rescued by BHS treatment. BHS treatment also regulated phosphorylation of p38, p53, ERK, and CREB. Therefore, our data indicated that BHS may reduce oxidative stress through regulation of ERK-CREB and p38-p53 signaling in the hippocampus, resulting in decreased neuronal damage and improved memory in rodent models of neurodegenerative disease.

## 1. Introduction

Alzheimer's disease (AD) is the most common neurodegenerative disease characterized by the gradual dysfunction of memory and cognition, which is in the form of adult-onset dementia [1]. Cholinergic dysfunction, defined by loss of cholinergic markers, occurs in the brain of AD patients and is related to loss of memory [2]. AD is associated with pathogenetic mechanisms such as excitotoxic events and free radical-induced oxidative stress [3, 4]. The pathological characteristics of AD brains include the presence of extensive oxidative stress and neuronal loss [5, 6]. Oxidative stress causes the accumulation of reactive oxygen species (ROS) in neurons, followed by damage to mitochondria and neuronal apoptosis [7]. Removal of ROS or prevention of their formation may delay the onset or slow the progression of AD via reduction of oxidative stress-mediated neuronal toxicity [5].

ROS can modulate the activation of mitogen-activated protein kinases (MAPK), such as extracellular signal-regulated kinase (ERK), and stress-activated kinases, such as p38, which regulate hippocampal synaptic plasticity [8]. ERK survival pathways are reported to be key signals that induce the cellular antioxidant mechanism and cAMP responsive element-binding protein (CREB) phosphorylation and regulate cell proliferation and survival [9–11]. It is known that p38 phosphorylates and activates the nuclear factor erythroid 2-related factor 2 (Nrf2) transcription factor [12, 13]. Nrf2 is then translocated into the nucleus, which in turn regulates the expression of certain antiapoptosis or oxidation genes [14, 15]. Cellular stress is well known to activate p53 and subsequently affect metabolism, oxidative stress, DNA repair, senescence, and apoptosis [16].

Scopolamine-treated animals are commonly used as a behavioral model to study AD and identify therapeutic targets for AD. This compound, which is an acetylcholine agonist, acts on nonselective muscarinic acetylcholine (ACh) receptors related to memory. Scopolamine-treated animals reveal several features of AD such as oxidative damage, mitochondria dysfunction, and A*β* pathology, followed by memory and cognitive deficits [17]. Excessive release of glutamate, which is another neurotransmitter, induces excitotoxicity and leads to oxidative stress and cell death [18].

Banhasasim-tang (BHS) is a traditional East Asian treatment as decoction including seven herbs. BHS is currently prescribed for the treatment of functional dyspepsia [19, 20] and has an antiapoptotic effect on chronic acid reflux esophagitis [21]. We recently demonstrated that BHS suppresses neuroinflammation in lipopolysaccharide-induced cognitive impairment [22]; however, the neuroprotective effect of BHS under oxidative stress conditions or its mechanisms are not well understood. Therefore, we hypothesized that BHS could be an antioxidant candidate to improve cognition in neuropathological conditions.

In the present study, we investigated if BHS administration improved spatial memory and reduced cell death in the hippocampus of scopolamine-treated mice. In addition, in an in vitro study, we explored if BHS treatment decreased cell apoptosis and ROS production in hippocampal cells through regulation of ERK and p38 signaling against oxidative stress.

## 2. Materials and Methods

### 2.1. Materials and Reagents

Powdered BHS was obtained from Hanpoong Pharm. Co Ltd. (Wanju, Korea). The herbal ingredients and composition ratio of the BHS decoction are indicated in Table 1. C57BL6/J mice were purchased from Dooyeol Biotech (Seoul, South Korea). Scopolamine, donepezil, Dulbecco's modified Eagle's medium (DMEM), L-glutamine, and glutamate were purchased from Sigma-Aldrich (St. Louis, MO, USA). Fetal bovine serum (FBS) and bovine serum albumin (BSA) were purchased from Hyclone (Logan, UT, USA). Penicillin/streptomycin was purchased from Gibco-Invitrogen (Carlsbad, CA, USA), and the cell counting kit-8 (CCK-8) was purchased from Dojindo Molecular Technologies (Kumamoto, Japan). The FITC Annexin V/propidium iodide (PI) apoptosis detection kit was purchased from BD Biosciences (San Diego, CA, USA). The Amplex Red Acetylcholine/Acetylcholinesterase Assay Kit and 5-(and-6)-carboxy-2′,7′-dichlorodihydrofluorescein diacetate (carboxy-H_2_DCFDA) were purchased from Invitrogen-Thermo Fisher (Pittsburgh, PA, USA). Various primary and secondary antibodies were acquired from Cell Signaling Technology (Danvers, MA, USA), Santa Cruz Biotechnology (Dallas, TX, USA), and Novus Biologicals (Minneapolis, MN, USA). Horseradish peroxidase- (HRP-) conjugated secondary antibodies were purchased from Bethyl (Farmingdale, NY, USA). Polyvinylidene fluoride (PVDF) membranes were purchased from Whatman (Piscataway, NJ, USA), and enhanced chemiluminescence (ECL) solution was purchased from Pierce (Rockford, IL, USA). The REAL EnVision detection system was purchased from DAKO Agilent (Santa Clara, CA, USA).

### 2.2. Preparation of BHS

BHS decoction powder (No. 18562) comprises *Pinellia ternata*, *Scutellaria baicalensis*, *Panax ginseng*, *Glycyrrhiza uralensis*, *Zingiber officinale*, *Coptis japonica*, and *Ziziphus jujuba*. We indicate the herbal composition ratio of BHS in Table 1. Dissolved BHS powder in water was used for this experiment.

### 2.3. Animals

**Figure 1:**
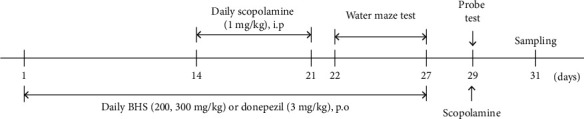
Overall experimental schedule. Mice were administered BHS (200 or 300 mg/kg) and donepezil (3 mg/kg) for 3 weeks daily. Scopolamine (1 mg/kg) was injected in mice at 14 days for 7 days. At 22 days, the MWM test was performed for 8 days, and treatment continued throughout the test. After behavioral testing, mice were sacrificed and tissue was harvested. BHS: Banhasasim-tang.

All animal experiments were approved by the Animal Care and Use Committee of Korea Institute of Oriental Medicine (approval number: KIOM-D-19-009). We purchased male C57BL/6J mice (5 weeks, 16–18 g) from Dooyeol Biotech (Seoul, South Korea) and housed them at a constant temperature (21 ± 2°C) and humidity with a 12 h light/dark cycle. We divided the 40 mice into five groups: CON (phosphate-buffered saline (PBS)+water), SCO (scopolamine+daily water), BHS 200 (scopolamine+daily BHS 200 mg/kg), BHS 300 (scopolamine+daily BHS 300 mg/kg), and DONE (scopolamine+daily donepezil 3 mg/kg). We injected the mice with scopolamine (1 mg/kg, i.p.) to induce memory impairment, whereas we injected the control mice with PBS after daily drug administration. The experimental schedule is represented in Figure 1.

### 2.4. Morris Water Maze

To test spatial memory, we performed the Morris water maze (MWM) test after administration of the drug, as described previously [22]. Briefly, we trained mice in a 1.2 m diameter pool filled with white water to reach a hidden platform (10 cm diameter) for 6 days. For each trial, we recorded the escape latency and distance using SMART video tracking software (Panlab, Barcelona, Spain). We conducted the probe test without the platform and recorded the time spent in the target zone (northeast quadrant).

### 2.5. Hematoxylin and Eosin (H&E) Staining

The mouse brains were removed and postfixed in a 10% formalin solution. Paraffin-embedded brains were sliced into 5 *μ*m thick coronal sections of the hippocampus region. Brain sections were incubated in xylene for 20 min twice and rehydrated through 100%, 90%, 80%, 70%, and 50% ethanol for 5 min. Sections were stained with H&E solution for 5 min and washed in distilled water. We dehydrated the sections in 50%, 70%, 90%, and 100% ethanol and cleared them in xylene for 20 min twice. The slides were mounted using mounting medium and imaged using a microscope (Olympus).

### 2.6. Acetylcholine (ACh) and Acetylcholinesterase (AChE) Activity

To measure neurotransmitter levels, we evaluated ACh expression in the hippocampus using the Amplex Red ACh/AChE Assay Kit (cat. A12217). Briefly, mouse hippocampi were lysed with RIPA buffer, and the same amount of protein was reacted in the working solution that consisted of the Amplex Red reagent, horseradish peroxidase (HRP), choline oxidase, and acetylcholinesterase for 90 min in the dark. Fluorescence was measured using a Spectra Max i3 (Molecular Devices). Results were expressed as the percentage of control cells.

### 2.7. Immunohistochemistry (IHC)

Slides were incubated in xylene for 20 min twice and rehydrated in 100%, 95%, 90%, 80%, 70%, and 50% ethanol. Slides were incubated in 10 mM sodium citrate buffer for 5 min and then in 3% H_2_O_2_ for 30 min in RT. After washing and blocking for 1 h with blocking solution, the sections were incubated with phospho-CREB (pCREB, #9198) overnight at 4°C and washed with PBS containing 0.1% Tween-20 (PBST). Slides were incubated with peroxidase-conjugated secondary antibody and detected with a REAL EnVision detection system (K5007). To stop detection, the sections were washed with PBST and dehydrated in 50%, 70%, 80%, 90%, 95%, and 100% ethanol, followed by xylene twice for 10 min. Slides were mounted and imaged at 10x magnification using a microscope (BX53F, Olympus Corporation, Tokyo, Japan). Lastly, pCREB positive cells were counted in the hippocampus region.

### 2.8. Cell Culture and Viability

HT22 cells were cultured in DMEM supplemented with 10% FBS and 1% penicillin/streptomycin in a 5% CO_2_ humidified incubator at 37°C. Cells were treated with BHS for 24 h, followed by cotreatment with 5 mM glutamate and BHS for 24 h. For the determination of cell viability, the CCK-8 assay was used after treatment with BHS and glutamate. CCK-8 solution was added and incubated for 2 h at 37°C. Absorbance was determined at 450 nm using a Spectra Max i3 (Molecular Devices, Sunnyvale, CA, USA). The results are expressed as a percentage of the control cells.

### 2.9. Annexin V/PI Apoptosis Assay

For the determination of cell apoptosis, flow cytometric analysis was performed using the FITC Annexin V/PI apoptosis detection kit. Cells were harvested and washed with phosphate-buffered saline (PBS). Binding solution containing 5% Annexin V and 5% PI solution was added to each sample and incubated for 20 min in the dark. Binding solution was added, and the samples were analyzed using a CytoFLEX Flow Cytometer (Beckman Coulter, Brea, CA, USA).

### 2.10. ROS Detection Assay

To determine ROS generation, cells were harvested after treatment and stained with 20 *μ*M carboxy-H_2_DCFDA (cat. C400) for 30 min at 37°C. The samples were analyzed using a CytoFLEX Flow Cytometer and imaged using a fluorescent microscope (ECLIPSE Ti2, Minato-ku, Tokyo, Nikon).

### 2.11. Western Blot

Mouse hippocampus tissue and cells were homogenized with RIPA lysis buffer and incubated for 20 min. Next, they were centrifuged at 12,000 RPM at 4°C for 20 min, and the supernatant was collected. An equal amount of proteins was quantified and loaded onto 10% sodium dodecyl sulfate polyacrylamide gel electrophoresis (SDS-PAGE) gel and then transferred to PVDF membranes. The membranes were blocked with 3% BSA solution and incubated with the following primary antibodies in 5% BSA solution: anti-*β*-actin (sc-47778), anti-Bcl-2 (#3498), pCREB (#9198), CREB (#9197), phospho-ERK (pERK, #9101), ERK (#9102), cleaved caspase-3 (#9664), HO-1 (#82206), phospho-p38 (pp38), MAPK (#9211), p38 MAPK (#9212), phospho-p53 (pp53, #2523), p53 (#2524), and Nrf2 (NBP1-32822) at 4°C overnight. Next, the membranes were washed with TBST and incubated with HRP-conjugated secondary antibodies (A90-137P or A120-108P) for 1 h at room temperature and then reacted with ECL solution (Pierce, Rockford, IL, USA). Immunoreactivity was recorded using a Q9 Alliance digital imaging system (UVITEC Ltd., England, UK). Results were quantified using ImageJ 1.52v software and normalized by *β*-actin or total form protein.

### 2.12. Data Analysis

All data were expressed as the mean ± standard error of the mean (SEM). Statistical analysis was performed using PRISM5 (GraphPad Software, San Diego, CA). Statistical comparisons of more than two groups were performed using one-way analysis of variance (ANOVA) and Student-Newman-Keuls post hoc test. A *p* value < 0.05 was considered to be statistically significant.

## 3. Results

### 3.1. Effect of BHS on Scopolamine-Induced Spatial Memory Decline in Mice

**Figure 2:**
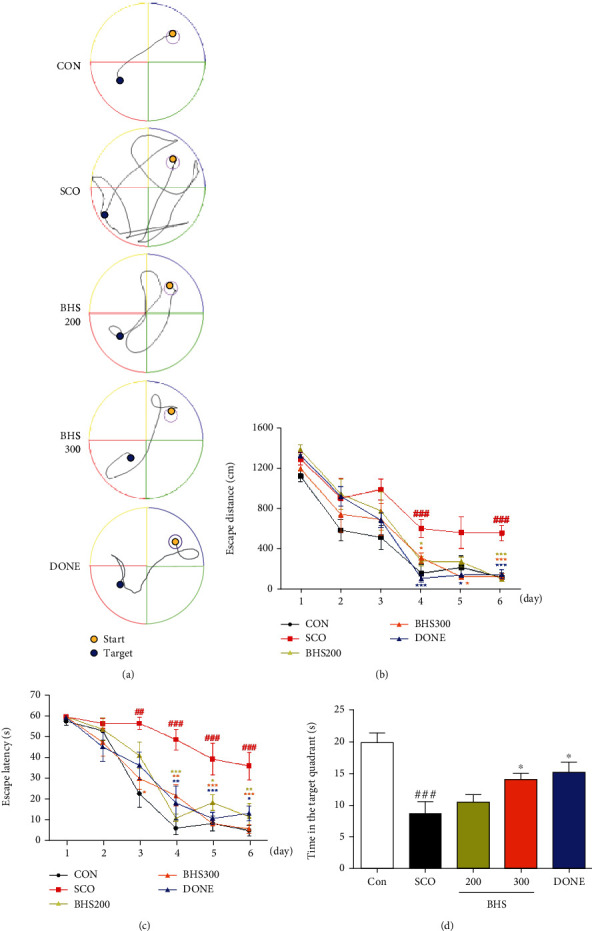
Effects of BHS on mouse memory loss induced by scopolamine in the Morris water maze test. (a) Representative swim paths on test day 6. (b) Swimming distance, (c) time spent to reach the target on days 1–6, and (d) the time spent in the target quadrant during the probe test. CON: saline (i.p.)+water (p.o.); SCO: SCO+water; BHS 200: SCO+BHS 200 mg/kg; BHS 300: SCO+BHS 300 mg/kg; DONE: SCO+donepezil 3 mg/kg. Mean ± SEM from *n* = 8 mice. ^##^*p* < 0.01 and ^###^*p* < 0.001 vs. CON; ^∗^*p* < 0.05, ^∗∗^*p* < 0.01, and ^∗∗∗^*p* < 0.001 vs. SCO by ANOVA with Newman-Keuls post hoc analysis. BHS: Banhasasim-tang; SCO: scopolamine; i.p.: intraperitoneal; p.o.: per oral.

To investigate whether BHS exerted a protective effect against scopolamine-induced spatial memory impairment, we performed the MWM test. We observed that the mice in the SCO group had a significantly increased time and distance to determine the platform compared with those in the CON group. Mice in the BHS 200, 300, and DONE groups showed significantly reduced time and distances compared to those in the SCO group (Figures 2(a)–2(c)). In addition, on the probe test, the time spent in the target quadrant was shorter for mice in the SCO group than those in the CON group. The times in the BHS 300 and DONE groups were significantly increased compared with the SCO group (Figure 2(d)). These results suggest that BHS treatment improved spatial memory in scopolamine-induced mice.

### 3.2. Effect of BHS on Acetylcholine Activity and Neuronal Death in the Brains of Scopolamine-Induced Mice

**Figure 3:**
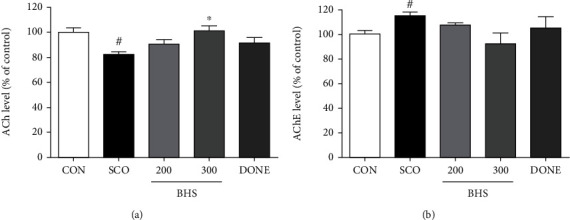
Effect of BHS on neurotransmitter expression in scopolamine-treated mice. (a) ACh level and (b) AChE activity in the hippocampus. Mean ± SEM from *n* = 4 mice. ^###^*p* < 0.001 vs. CON; ^∗^*p* < 0.05 vs. SCO by ANOVA with Newman-Keuls post hoc analysis. ACh: acetylcholine; AChE: acetylcholinesterase.

**Figure 4:**
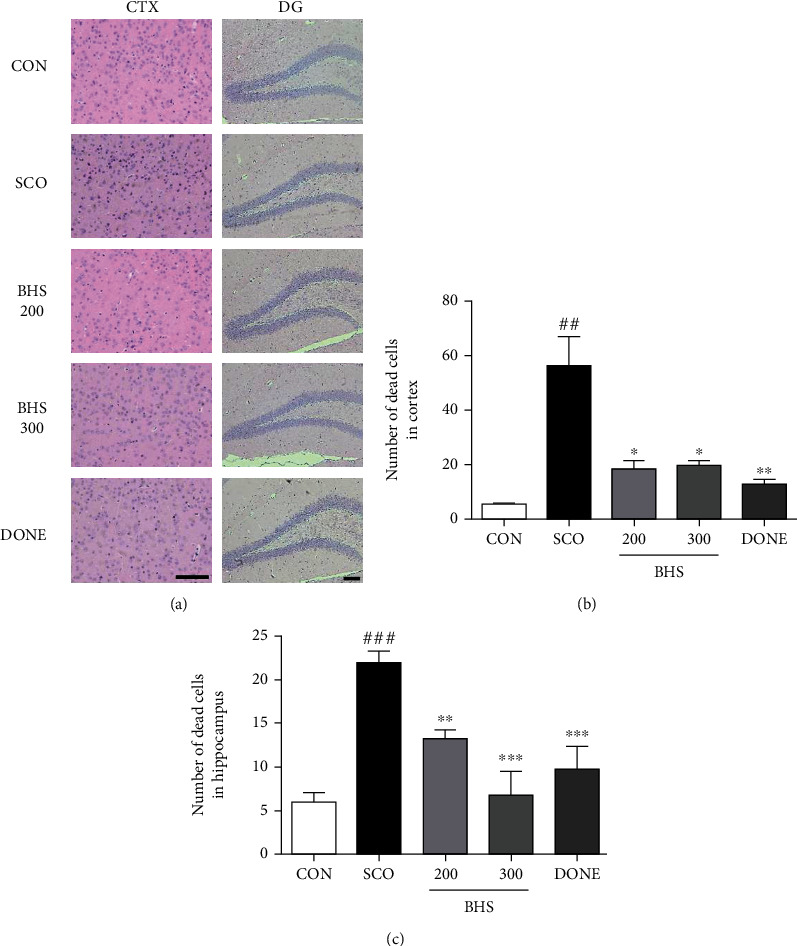
Effects of BHS on scopolamine-induced neuronal damage in mice. (a) H&E staining images and the number of dead neurons in the (b) hippocampus and (c) cortex of each group of mice. Mean ± SEM from *n* = 3. ^##^*p* < 0.01 and ^###^*p* < 0.001 vs. CON; ^∗^*p* < 0.05, ^∗∗^*p* < 0.01, and ^∗∗∗^*p* < 0.001 vs. SCO by ANOVA with Newman-Keuls post hoc analysis. Scale bars = 200 *μ*m. H&E: hematoxylin and eosin; CTX: cortex; DG: dentate gyrus.

**Figure 5:**
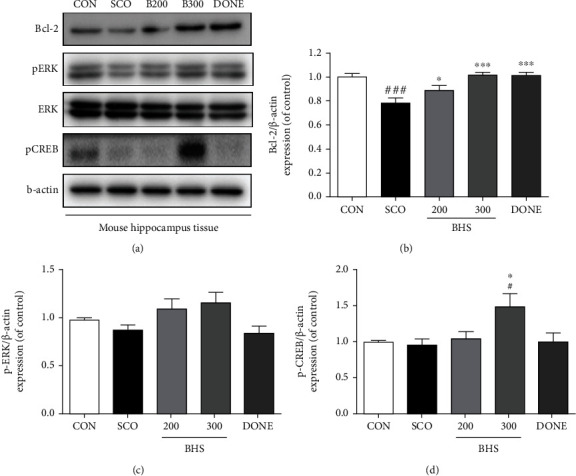
Effects of BHS on apoptotic and survival-related protein expression in the hippocampus. (a) Western blot and its histogram for (b) Bcl-2, (c) pERK/ERK ratio, and (d) pCREB. Histograms show protein relative to *β*-actin and total form protein. Mean ± SEM from *n* = 4. ^#^*p* < 0.05 and ^###^*p* < 0.001 vs. CON; ^∗^*p* < 0.05 and ^∗∗∗^*p* < 0.001 vs. SCO by ANOVA with Newman-Keuls post hoc analysis.

A previous study reported that mice injected with scopolamine show loss of acetylcholine activity and neurons [23]. Thus, we investigated acetylcholine activity in the hippocampus. Compared to the CON group, the ACh level in the SCO group was decreased; however, in the BHS 300 group, it was significantly increased (Figure 3(a)). Increased AChE levels in the SCO group tended to be reduced by BHS 300 mg/kg administration (Figure 3(b)). Additionally, we investigated the protective effects of BHS against scopolamine-induced neuronal loss. As shown by H&E staining of the brain, the number of dead neurons in the SCO group was significantly increased compared to that in the CON group in the cortex and hippocampus. Compared to the SCO group, the number of dead cells in the BHS 200, BHS 300, and DONE groups was significantly reduced (Figures 4(a)–4(c)). We also checked the expression of Bcl-2, a protein related to apoptosis, in the hippocampus. Bcl-2 expression in the SCO group was decreased compared to that in the CON group; however, the protein level in the BHS 300 and DONE groups was significantly increased compared to the SCO group (Figures 5(a) and 5(b)). These results suggest that BHS treatment restored acetylcholine activity and protected neuronal cells in the hippocampus of scopolamine-induced mice.

### 3.3. BHS Regulates ERK and CREB Signaling in the Hippocampus of Scopolamine-Induced Mice

**Figure 6:**
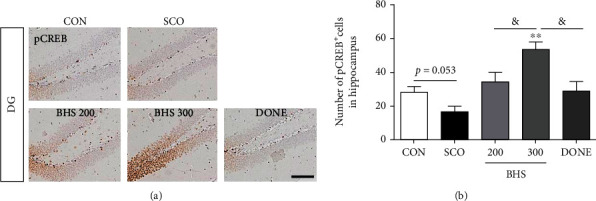
Immunostaining of pCREB in the hippocampus and cortex. (a) Microphotographs and (b) histogram for pCREB in the hippocampus. Mean ± SEM from *n* = 3. ^#^*p* < 0.05 vs. CON; ^∗∗^*p* < 0.01 vs. SCO; ^&^*p* < 0.05 vs. BHS 300 by ANOVA with Newman-Keuls post hoc analysis. Scale bars = 200 *μ*m. DG: dentate gyrus.

ERK and CREB signaling is related to memory in the hippocampus [24]. We investigated whether BHS increases ERK-CREB signaling in the hippocampus of scopolamine-induced mice. We assessed protein levels of pERK and pCREB using Western blot. We found that the decreased pERK expression induced by scopolamine treatment was elevated by BHS treatment, but not significantly (Figure 5(c)). Compared to the CON and SCO groups, markedly increased expression of pCREB protein in the BHS 300 group was observed (Figure 5(d)). We performed IHC staining to confirm the histological expression of pCREB in the hippocampus. A decreased number of pCREB positive cells in the SCO group were observed, and the cell number in the BHS 300 group was significantly increased compared with that of the other groups (Figure 6). In the DONE group, expression of pERK and pCREB protein was similar to that in the SCO group. These results suggest that BHS treatment regulated ERK and CREB signaling in the hippocampus of scopolamine-induced mice.

### 3.4. Effects of BHS on Glutamate-Induced Cytotoxicity in Mouse Hippocampal Cells

**Figure 7:**
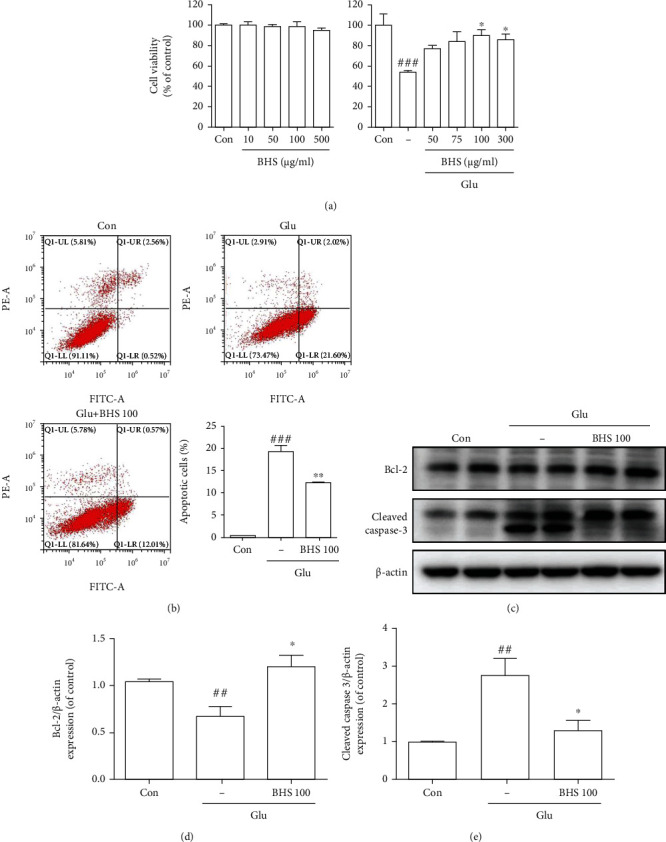
Protective effects of BHS on glutamate-induced hippocampal cells. (a) Cell viability and (b) representative flow cytometry analysis scattergram and histogram for Annexin V/PI staining. Quantitative analysis of the histograms is expressed as the percentage of apoptotic cell death. (c) Western blot and its densitometric analysis for (d) Bcl-2 and (e) cleaved caspase 3. Mean ± SEM from three independent experiments. ^#^*p* < 0.05, ^##^*p* < 0.01, and ^###^*p* < 0.001 vs. CON; ^∗^*p* < 0.05, ^∗∗^*p* < 0.01, and ^∗∗∗^*p* < 0.001 vs. Glu. Glu: glutamate.

To confirm whether BHS protects hippocampal neuronal cells, we performed a CCK-8 assay. To assess the protective effect of BHS in hippocampal cell-derived lines, BHS pretreatment was performed and the CCK assay was used to confirm cell viability. We observed no toxicity at 10–500 *μ*g/ml concentrations of BHS treatment (Figure 7(a), left). Cell survival in glutamate-treated cells was reduced compared with untreated cells and increased in a concentration-dependent manner with BHS treatment compared with glutamate-treated cells (Figure 7(a), right). We observed that the most effective concentration was 100 *μ*g/ml. As a result, the following experiments were performed using this dose. These results suggest that BHS treatment protected neuronal cells against glutamate-induced toxicity.

### 3.5. Effects of BHS on Glutamate-Induced Apoptosis in Mouse Hippocampal Cells

To determine whether BHS decreases glutamate-induced apoptosis in hippocampal cells, we performed flow cytometry. The number of apoptotic cells in glutamate-treated cells was increased compared to control cells, and the number of cells was markedly decreased by BHS treatment (Figure 7(b)). To assess whether apoptosis-related protein was regulated by BHS treatment, we assessed the expression level of Bcl-2 and cleaved caspase-3 (Figures 7(c)–7(e)). Antiapoptotic protein Bcl-2 expression was significantly decreased in glutamate-treated cells compared to control cells, and BHS treatment significantly increased Bcl-2 expression (Figure 7(d)). We also observed an increase in the expression of cleaved caspase 3, an apoptotic protein, in glutamate-treated cells, and expression of this protein was significantly reduced by BHS treatment (Figure 7(e)). These results suggest that BHS treatment reduced apoptosis in hippocampal neuronal cells.

### 3.6. Effects of BHS in Glutamate-Induced Oxidative Stress in Mouse Hippocampal Cells

**Figure 8:**
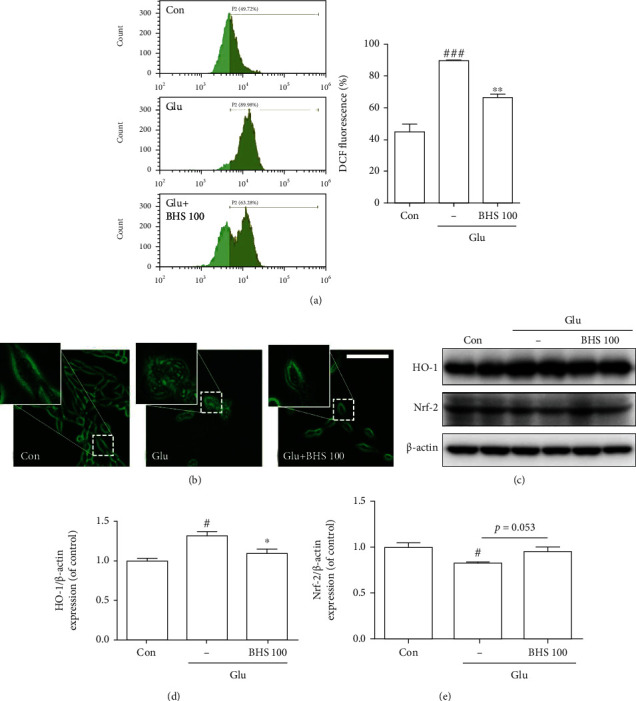
Effects of BHS on ROS production in glutamate-induced hippocampal cells. (a) Representative flow cytometry analysis for carboxy-H_2_DCFDA and quantitative analysis of the histograms expressed as the percentage of ROS production. (b) Photomicrographs for DCFDA expression in cells. (c) Western blot band and its densitometric analysis for (d) HO-1 and (e) Nrf2. Mean ± SEM from three independent experiments. ^#^*p* < 0.05 and ^###^*p* < 0.001 vs. CON; ^∗^*p* < 0.05 and ^∗∗^*p* < 0.01 vs. Glu. Glu: glutamate. Scale bars = 100 *μ*m.

To investigate the effects of BHS against oxidative stress, ROS generation was evaluated using carboxy-DCFDA. DCF fluorescence in glutamate-treated cells increased significantly compared to control cells, and BHS treatment has reduced an increasing fluorescence by glutamate treatment (Figure 8(a)). We observed increased ROS accumulation in the cell cytosol of glutamate-treated cells, and the increased ROS was markedly reduced by BHS treatment on morphological analysis (Figure 8(b)). Protein expression of HO-1, an oxidative stress marker, was significantly increased by glutamate treatment and significantly decreased by BHS treatment (Figure 8(d)). The expression of Nrf2 was significantly reduced in glutamate-treated cells and increased by BHS treatment (Figure 8(e)). These results suggest that BHS treatment reduced overproduction of ROS in hippocampal cells.

### 3.7. BHS Regulates the Expression of p38, p53, ERK, and CREB in Mouse Hippocampal Cells

**Figure 9:**
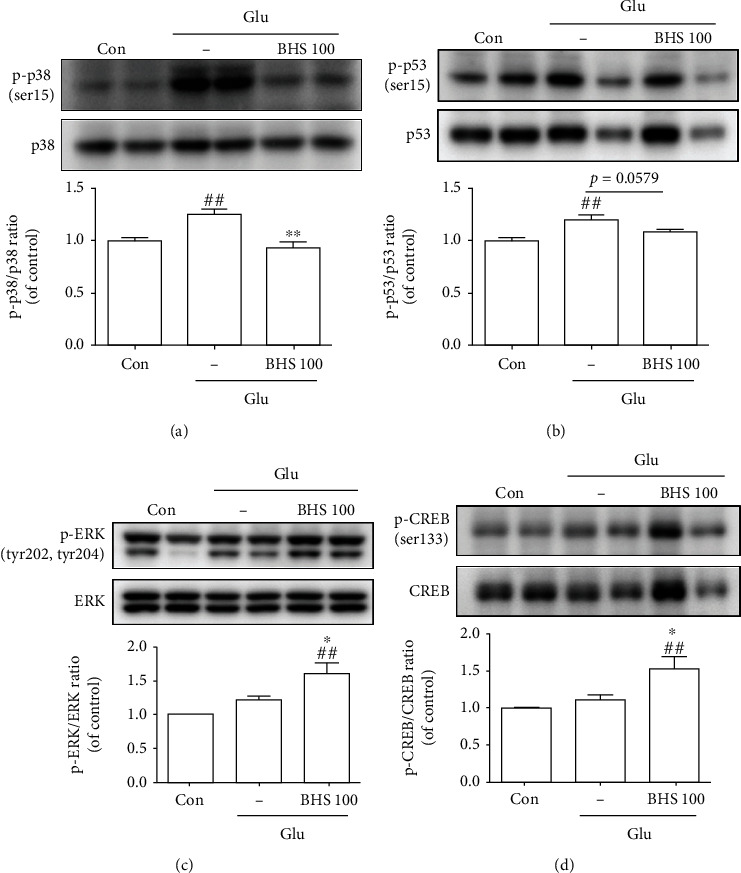
BHS regulates p38, p53, ERK, and CREB expression in glutamate-induced hippocampal cells. Western blot and its densitometric analysis for (a) p-p38/p38, (b) p-p53/p53, (c) pERK/ERK, and (d) pCREB/CREB ratio. Mean ± SEM from three independent experiments. ^##^*p* < 0.01 vs. CON; ^∗^*p* < 0.05 and ^∗∗^*p* < 0.01 vs. Glu. Glu: glutamate.

A previous study showed that p38, p53, ERK, and CREB activation reduced ROS production [25]. Therefore, we investigated whether BHS treatment regulates the activation of these signaling molecules (Figure 9). The p-p38/p38 and p-p53/p53 ratios were significantly decreased by glutamate treatment compared to those in the control cells, and these ratios were increased by BHS treatment (Figures 9(a) and 9(b)). The pERK/ERK and pCREB/CREB ratios were significantly increased in BHS-treated cells compared with control and glutamate-treated cells (Figures 9(c) and 9(d)). These results suggest that BHS treatment regulated the activation of p38, p53, ERK, and CREB in hippocampal cells.

## 4. Discussion

BHS is used for treating dyspepsia as a traditional decoction [20]. Our previous studies showed that BHS treatment attenuates neuroinflammation and cognitive dysfunction [22]. However, the effects of BHS on oxidative stress-induced neuronal damage remain largely unknown. In the present study, we explored the neuroprotective effect of BHS on attenuating oxidative stress and cell death and the mechanism underlying the protection of neuronal cells by BHS against oxidative stress in vivo/in vitro.

Oxidative stress and cholinergic dysfunction play a crucial role in cognitive impairment [6]. The administration of scopolamine induced oxidative stress, apoptosis, and dysfunction of the cholinergic system in the brain, followed by memory impairments [4, 23]. In our study, BHS administration improved spatial memory function (Figure 2) and promoted neuronal survival and cholinergic activity in the scopolamine-induced mouse (Figures 3 and 4).

A previous study confirmed the involvement of the ERK-CREB signaling pathway in neuroprotection in hippocampal neurons after exposure to ischemia [24, 26]. CREB phosphorylation by multiple protein kinases such as ERK is involved in nerve cell excitation, memory formation, and neuroprotection [27, 28]. In our study, BHS administration increased the expression of the antiapoptotic protein Bcl-2 and phosphorylation of ERK and CREB in the hippocampus of scopolamine-induced mice. Interestingly, donepezil treatment influenced the expression of the antiapoptotic protein without changing ERK/CREB signaling (Figures 5 and 6). The most commonly used drug for clinical AD is donepezil, which is a potent and rapidly reversible inhibitor of AChE that improves cognition. Our findings support that BHS enhances acetylcholine activity and activates neuroprotective action through endogenous regulation of ERK/CREB signaling.

To investigate signaling-related neuroprotection of BHS against oxidative stress in hippocampal neuronal cells, we explored using glutamate-induced excitotoxicity. Excessive secretion of glutamate from the central nervous system to the outside of neurons induced typical excitotoxicity, which in turn promotes apoptosis due to oxidative stress [30]. It is an important feature in neurodegenerative processes, including AD. Our study showed that BHS treatment reduces apoptosis and death of hippocampal cells (Figure 7).

Glutamate excitotoxicity also induces the production of ROS in neurons [29, 30]. This toxicity is exerted by depletion of glutathione (GSH), leading to free radical accumulation in the HT22 cell line [17], and depletion of cellular GSH triggers HO-1 expression [31]. A previous study showed that HO-1 is increased in the early stages of oxidative stress, followed by increased apoptosis factors [32]. In our in vitro model, HO-1 expression was increased in glutamate-induced excitotoxicity (Figures 8(c) and 8(d)).

Nrf2 participates in defense against oxidative stress and plays an essential role in neuroprotection by regulating the expression of antioxidant molecules and enzymes [33]. Nrf2 proteins are bound to Kelch-like ECH-associated protein 1 (Keap1). In response to oxidative stress, Nrf2 protein can dissociate from Keap1, following transfer from the cytoplasm to the nucleus, and lead to the transcription of antioxidant genes [14, 15]. Although the level of Nrf2 protein expression in whole cells was assessed in this study, we did not explore how Nrf2 translocated into the nucleus and was activated, which is a limitation of our study. However, previous studies have reported that oxidative stress regulates the interaction between Nrf2 and Keap1 and that Nrf2 activation is dependent on increasing Nrf2 protein stability [34]. It has also been reported that antioxidants such as ascorbic acid and butyl hydroxyanisole increased total Nrf2 protein, which can sense antioxidant activities [35]. Similarly, we observed that total Nrf2 protein expression was reduced under glutamate-induced oxidative stress and was recovered by BHS treatment (Figure 8). Therefore, BHS reduced ROS production through regulation of Nrf2 expression against glutamate-induced oxidative stress in hippocampal cells.

p38 and ERK contribute to cell apoptosis and survival [36, 37], which are regulated by oxidative stress [25]. ROS accumulation activates p38 and leads to p53 activation [38], which is involved in apoptosis [16, 39]. ERK acts as a negative feedback loop, suppressing p53-induced apoptosis [40] and inducing CREB phosphorylation, which plays a role in cellular antioxidant action and antiapoptosis [9, 41]. In addition, Nrf2 signaling is modulated by ERK and p38 [42, 43]. Our results revealed that BHS treatment suppresses the activity of p38-p53 signaling and increases the activity of ERK-CREB signaling (Figure 9). Our findings therefore provide evidence to support that BHS activates ERK and p38 signaling, which modulates CREB and p53 signaling and attenuates oxidative stress-mediated neuronal cell death in the hippocampus.

## 5. Conclusion

In summary, we demonstrated that BHS recovered spatial memory and protected neuronal cells against scopolamine-induced damage in vivo. In hippocampal cells, BHS attenuated cell apoptosis and oxidative stress by increasing the antioxidant protein Nrf2 and the survival signal CREB. These antioxidant actions of BHS were due to phosphorylation of ERK and p38. Overall, BHS exhibits neuroprotective effects against oxidative stress-mediated neuronal cell death, suggesting that it might be used to treat neurodegenerative disease-associated cognitive impairment, such as AD.

## Figures and Tables

**Table 1:** Composition ratio of Banhasasim-tang (BHS).

Scientific name	Parts	Composition ratio (%)
*Pinellia ternata* (Thunb.) Makino	Tuber	24.5
*Scutellaria baicalensis* Georgi	Root	14.6
*Panax ginseng* C.A. Mey	Root	14.6
*Glycyrrhiza uralensis* Fisch	Root and rhizome	14.6
*Zingiber officinale* Roscoe	Rhizome	12.2
*Coptis japonica* (Thunb.) Makino	Rhizome	4.6
*Ziziphus jujuba* Mill	Fruit	14.6

## Data Availability

The data that support the findings of this study are available on request from the corresponding author.
